# Acute coagulopathy in trauma: with or without shock? That is the question

**DOI:** 10.1186/cc13931

**Published:** 2014-06-19

**Authors:** Juan José Egea-Guerrero, Ana Rodríguez-Rodríguez, María Dolores Freire-Aragón

**Affiliations:** 1 NeuroCritical Care Unit, Virgen del Rocío University Hospital, IBiS/CSIC/University of Seville, Avda. Manuel Siurot s/n 41013, Seville, Spain

## 

In an interesting and original article in a recent issue of *Critical Care*, Oshiro and colleagues describe the profile of acute coagulopathy of trauma-shock (ACOTS) using viscoelastic techniques [1]. The authors, in light of their results, demonstrate the hypothesis that ACOTS is a type of disseminated intravascular coagulation (DIC) with a fibrinolytic profile. However, we would like to highlight some aspects we consider to be of interest. It has caught our attention that, fulfilling national laws, patients were not subjected to fluid resuscitation during transport to the hospital. We understand that aggressive fluid resuscitation is deleterious to the patient and induces the possibility of dilutional coagulopathy [2]–[4]. However, despite the absence of strong evidence in this respect, current guidelines recommend its administration in patients with hemodynamic compromise after trauma but during the prehospital phase [4,5]. In this regard, we think the submission of information related to the hemodynamic status of patients during this phase or at hospital admission would have been interesting. It is widely known that trauma hypotension induces hyperfibrinolysis increase, which finally promotes ACOTS. Therefore, the lack of such data prevents us from knowing, from a physiological point of view, whether the increase of DIC profile with hyperfibrinolytic character was enhanced by these motifs [2]. Regardless of these observations, we consider that the study presents important evidence for understanding the ACOTS in trauma patients, and this is one of the major challenges to overcome during the management of these patients.

## Author’s response

Satoshi Gando

We appreciate the interest of Egea-Guerrero and colleagues in our article regarding DIC in trauma [1]. We agree that the patient’s hemodynamic status during transport and at hospital admission is important. Although we did not collect data for hemodynamic parameters, we presented direct evidence of hypoperfusion based on the lactate levels in the subjects who developed DIC. Our previous study demonstrated that DIC occurs during the early phase of trauma independently of lactate levels [6]. Trauma itself can cause DIC, whereas hypoperfusion accelerates DIC via release of tissue plasminogen activator from endothelial cells, resulting in the fibrinolytic phenotype. ACOTS has recently attracted much attention in the field of trauma. However, many questions have been raised, as indicated in ‘ACOTS: with or without shock? That is the question’. The definition of ACOTS requires the inclusion of shock-induced hypoperfusion, increased activated protein C (APC) level, APC-mediated thrombin shutoff and plasminogen activator inhibitor-1 (PAI-1) inhibition in the circulation, the presence of normal endothelial cells that can newly express endothelial thrombomodulin, and soluble thrombomodulin with full domains and 100% activity. However, the APC levels did not increase in ACOTS patients and no differences have been reported in thrombin generation or the PAI-1 levels between ACOTS patients and control subjects [7]. Therefore, ‘ACOTS: reality or myth? That is the question’ would also be considered a suitable title. Regardless of whether ACOTS is a reality or a myth, the points raised in their letter are very important for trauma management.

## Abbreviations

ACOTS: Acute coagulopathy of trauma-shock; APC: Activated protein C; DIC: Disseminated intravascular coagulation; PAI-1: Plasminogen activator inhibitor-1.

## Competing interests

The authors declare that they have no competing interests.
